# Effects of *Aspergillus niger* on cyanogenic glycosides removal and fermentation qualities of ratooning sorghum

**DOI:** 10.3389/fmicb.2023.1128057

**Published:** 2023-02-20

**Authors:** Jianrong Zhai, Bo Wang, Yingpeng Sun, Jianfeng Yang, Junfeng Zhou, Tianyu Wang, Wenlan Zhang, Cai Qi, Yanjun Guo

**Affiliations:** ^1^Qingdao Key Laboratory of Specialty Plant Germplasm Innovation and Utilization in Saline Soils of Coastal Beach, Qingdao Agricultural University, Qingdao, China; ^2^Key Laboratory of National Forestry and Grassland Administration on Grassland Resources and Ecology in the Yellow River Delta, Qingdao Agricultural University, Qingdao, China; ^3^College of Animal Science and Technology, Southwest University, Chongqing, China; ^4^College of Grassland Science, Qingdao Agricultural University, Qingdao, China; ^5^Chongqing Jiangxiaobai Farm Co., Ltd., Chongqing, China

**Keywords:** *Aspergillus niger*, cyanogenic glycosides, hydrocyanic acid, ratooning sorghum, silage

## Abstract

**Introduction:**

Cyanogenic glycosides (CNglcs) are bioactive plant products involving in plant defense against herbivores by virtue of their abilities to release toxic hydrogen cyanide (HCN). *Aspergillus niger* has been shown to be effective in producing *β*-glucosidase, which could degrade CNglcs. However, whether *A. niger* could remove CNglcs under ensiling conditions is still unknown.

**Methods:**

In this study, we first investigated the HCN contents in ratooning sorghums for two years, then the sorghums were ensiled with or without the addition of *A. niger*.

**Results:**

Two years’ investigation indicated that the contents of HCN in fresh ratooning sorghum were larger than 801 mg/kg FW (fresh weight), which could not be reduced by silage fermentation under safety threshold (200 mg/kg FW). *A. niger* could produce *β*-glucosidase over a range of pH and temperature, which degraded the CNglcs and removed the hydrogen cyanide (HCN) at early days of ratooning sorghum fermentation. The addition of *A. niger* (2.56 × 10^7^ CFU/ml) altered the microbial community, increased bacterial diversity, improved the nutritive qualities, and reduced the HCN contents in ensiled ratooning sorghum lower than 100 mg/kg FW after 60 days of fermentation. Overall, the addition of 150 ml *A. niger* + 50 ml sterile water per 3 kg silage could efficiently remove CNglcs from ratooning sorghum silage.

**Conclusion:**

In conclusion, *A. niger* could produce *β*-glucosidase which degraded the CNglcs during the early days of fermentation, benefiting the ensiling process and improving the utilization of ratooning sorghum.

## 1. Introduction

Cyanogenic glycosides (CNglcs) are bioactive plant products derived from amino acids (Cowan et al., 2021), which involve in plant defense against herbivores by virtue of their abilities to release toxic hydrogen cyanide (HCN) upon tissue disruption (Picmanova et al., 2015). These compounds are widely distributed in plants such as sorghum, cassava, lima beans, bamboo shoots, spinach, and fruits such as drupes, pears and pip (Codex Committee on Contaminants in Foods, 2008). Though CNglcs are not toxic to plant itself, its release of HCN may lead to the risk of acute cyanide poisoning in humans or livestock (Moller, 2010). Overall, 200 mg/kg fresh weight (FW) of HCN in forage is regarded as the safety threshold for livestock (Kumari et al., 2021), and up to 50% of the ingested CNglcs may result in human cyanide exposure (Carlsson et al., 1999). Therefore, release of HCN from CNglcs before they are delivered into animal gut will greatly reduce toxic risk.

Structurally, CNglcs includes a core carbon attached to a CN moiety and two substituent groups attached to a sugar by a glycosidic bond. Due to the difference of the substituent groups, the CNglcs differ among plant species, such as Duhrrin in sorghum, Linamarin in cassava, Prunasin in ferns, and Taxiphyllin in bamboo shoots (Cressey and Reeve, 2019). Irrespective of CNglcs structures, release of HCN from CNglcs can occur through enzymatic hydrolysis by *β*-glycosidase, where the glycoside is enzymatically converted to the corresponding cyanohydrin, followed by spontaneously decomposing to form HCN (Wanasundara et al., 1993). Though *β*-glycosidases are widely distributed in seeds and microbes, their activities are depending upon the concentrations of substrates and the environmental conditions (Briante et al., 2001; Michlmayr et al., 2012). Therefore, suitable food process conditions might benefit the activities of *β*-glycosidases, improving the release of HCN.

Ensiling is one of the important methods widely used in forage production (Yang et al., 2019). Previous study indicated that ensiling ground pods of *Acacia sieberiana* for 45 days removed 86.1% of CNglcs (reaching the non-toxic levels for livestock) (Ngwa et al., 2004). However, in another study, although the HCN contents of cassava roots was reduced from 506.8 mg/kg (FW) to 295.8 mg/kg (FW) by silage, the final HCN contents in silage was still above the safety threshold (200 mg/kg FW) (Chander et al., 2004). This implied that ensiling benefited the release of HCN but the activities of *β*-glycosidases might not be high enough to remove the CNglcs under safety level. The ensiling is processed under anaerobic conditions, with the pH value decreasing with the extension of fermentation, which might limit the enzymatic activity of *β*-glycosidase as well as the activity of microorganisms producing *β*-glycosidases. Therefore, detailed exploration of the enzymatic activity of *β*-glycosidase under various environments is needed.

*Aspergillus niger* is a haploid filamentous fungus widely distributed in various environments (Yu et al., 2021). As an aerobic microorganism, *A. niger* is the source of several bioactive compounds and industrial enzymes, as well as additives for waste management and biotransformations (Hasanuzzaman et al., 2014). Interestingly, inoculation of *A. niger* strain had positive effect on the removal of CNglcs from rubber seed meal by fermentation (Liu et al., 2010). This implied that *A. niger* might be used in silage fermentation to degrade the CNglcs. However, whether the low pH levels and anaerobic conditions of silage will benefit CNglcs removal and how the *A. niger* remove the CNglcs are still not clear.

To fill the gap between the removal rate of CNglcs and the addition of *A. niger* in silage production, we evaluated the changes of HCN contents in ensiled ratooning sorghum with the addition of different concentrations of *A. niger*. Ratooning sorghums regrow from the dormant axillary buds on the sorghum stem nodes and tillers from the stem base nodes, or sprouts branches from the upper part of the plant. For some sorghum varieties, when the straws are harvested, the sorghum will regrow, and even can be harvested for cereal during late season when the environmental conditions are suitable. However, under most conditions, the temperature is not suitable for growth, or the seeding date of the following crop cannot guarantee the maturation of sorghum. Therefore, the ratooning sorghums have to be harvested in their vegetative stages or in their blossom or filling stages, which has the potential to be used as fodder or silage. Currently, these regrown sorghums are directly returned to soils (Seglah et al., 2020), or used as cooking fuel. Due to the risk of HCN poison, the ratooning sorghum are seldomly used as feeds. For some cultivars of sweet sorghum (Mule 8,000), the HCN contents reached 482 mg/kg at early growing stages (Shehab et al., 2020). Drought increased the foliar HCN contents in sorghum from 709 mg/kg in well-watered plants to 1,155 mg/kg in drought-stressed plants (Gleadow et al., 2016). Therefore, reducing the HCN contents in sorghum silage might improve their utilizations. We hypothesized that adding *A. niger* to silage could remove the CNglcs to safety threshold. The following three research aspects were mainly addressed, (1) to prove the efficiency of *A. niger* addition in removing CNglcs; (2) to elucidate the mechanism of *A. niger* in removing CNglcs; and (3) to evaluate the potential utilization of ratooning sorghum as safe feeds.

## 2. Materials and methods

### 2.1. Experimental site and materials

The experiment was conducted at Jiangxiaobai Red Sticky Sorghum Planting Demonstration Base (29°07′N, 106°16′E), located in Chongqing, China. The area has a mean temperature of 17.9°C, and a mean annual rainfall of 1,100 mm during the last 30 years (Chongqing Meteorological Bureau). The soil was a neutral yellow soil with a pH 7.5, alkali-dispelled nitrogen 96.15 mg/kg, available phosphorus (Olsen-P) 17.83 mg/kg, and available potassium 90.15 mg/kg.

The red sticky *Sorghum bicolor* cv. Chuannuoliang 2 is the principal variety planting in this base for Baijiu (distilled liquor) production. Sorghum-Canola rotation system is used in this base. Generally, the sorghum will be sown in May after the canola is harvested, and be harvested in August, then the canola will be sown in October. From August to October, there is about 2 months’ fallow period, which is suitable for the regrowth of the harvested sorghum, called ratooning sorghum. Currently, the ratooning sorghum is chopped and returned to soils or discarded. In this study, on 2th October, 2020 and 10th October, 2021, the ratooning sorghum at their heading and flowering stage were harvested for whole plant silage (above 5 cm from the ground). These ratooning sorghums have the potential to be used as fodder or silage.

*A. niger* was bought from Beijing Beina Chuanglian Biotechnology Research Institute. Before used as additives, the *A. niger* was cultured on Potato Dextrose Agar medium (PDA) at 28°C for 72 h, then, the activated *A. niger* was inoculated into Potato Dextrose Broph liquid medium (PDB) at 32°C for 3 days at 170 ~ 180 r/min (Wang et al., 2007). When the concentration of *A. niger* solution reached 2.56 × 10^7^ CFU/mL, it was evenly sprayed on the silage as additives.

### 2.2. Determination of agronomic characters

One day before the harvest of the ratooning sorghum, ten sorghum plants were randomly sampled from the fields to measure the agronomic characters, including the plant height, stem diameter, stem leaf ratio, leaf number, and fresh plant yields.

### 2.3. Experimental design

In experimental one, the harvested ratooning sorghums (2020) were kept under room temperature for 12 h until the water contents reached *ca.* 78%. Then, the whole plants were chopped into *ca.* 2 cm length, thoroughly mixed, and ensiled without additives (Control), with adding 1 g/kg of cellulase, and with adding 1 g/kg of xylanase. The samples were packed into polyethylene plastic bags (18 cm × 26 cm), vacuum sealed, and stored at room temperature (~25°C) for 7, 15, 30 and 60 days. Each silage bag contained 500 g samples and each treatment replicated three times.

In experimental two, the harvested ratooning sorghums (2021) were used. The whole plants were chopped and ensiled with 200 ml sterile water / 3 kg (Control), with 50 ml *A. niger* + 150 ml sterile water / 3 kg (AN50), with 100 ml *A. niger* + 100 ml sterile water / 3 kg (AN100), with 150 ml *A. niger* + 50 ml sterile water / 3 kg (AN150), and with 200 ml *A. niger*/3 kg (AN200). The silage bags were placed at room temperature (~ 25°C) for 48 h before sealing, to provide oxygen for *A. niger* propagation (Zhou et al., 2019). The starting time of the fermentation was calculated at the sealing. The other ensiling procedures were same as the experimental one.

In experimental three, the activated *A. niger* was inoculated into PDB at 32°C for 3 days at 170 ~ 180 r/min. When the concentration of *A. niger* solution reached 2.56 × 10^7^ CFU/mL, the *A. niger* was added into fermentation medium. A single-factor experiment was designed with different inoculation concentrations of *A. niger* (50, 100, 150, and 200 mL of *A. niger* per 200 mL sterile water), pH levels (3.0–9.0), and temperatures (20, 25, 30, 35 and 40°C). Each treatment replicated four times.

### 2.4. HCN content determination in fresh ratooning sorghum and silage

Ten-gram dry samples were taken and homogenized in 500 mL distilling bottle with 200 mL distilled water, 20 mL of zinc acetate and 2 g of tartaric acid. The lower end of the condenser tube was inserted into a 100 mL conical flask with 20 g/ml sodium hydroxide solution, to collect the released HCN gas. This was done for three times until the volume of distillate reached *ca.* 250 mL. Then, 10 mL distillate were taken and 1 mL of 10 g/mL sodium hydroxide solution and a drop of phenolphthalein indicator were added, and adjusted with acetic acid solution until the red color fades. Next, 5 mL phosphoric buffer were added and bathed at 37°C for 10 min, and then 0.25 mL chloramine T solutions were added and stand for 5 min. Finally, 5 mL isonicotinic-pyrazolone solution were added to a final volume of 25 mL, water bathed at 37°C for 40 min, and then the OD value was measured at 638 nm (Dominguez et al., 2002).

### 2.5. Activities of *β*-glucosidase produced by *A. niger*

After 0, 12, 24, 48, 72, and 96 h of cultivation, the fermentation medium was centrifuged at 3000 r/min for 10 min to obtain the supernatant crude enzyme solution. Then, 0.1 mL of the enzyme solution and 0.9 mL of 0.2 mol/L Na_2_HPO_4_–0.1 mol/L citric acid buffer solution with pH 4.5 were added, and water bathed at 50°C for 10 min. Next, 1 mL of 5 mmol/L P-NPG solution preheated for 10 min was added. Subsequently, 1 mL of 1 mol/L Na_2_CO_3_ solution was added to stop the reaction. Finally, the extract was allowed to stand for 5 min to measure the OD value at 400 nm (Li et al., 1998). The activity of *β*-glucosidase was calculated as follows:


U=C/(10×0.1)×N=C×N

Note: *U* is enzyme activity (u/mL); 10 is reaction time; *N* is dilution multiple of original enzyme solution; 0.1 is take 0.1 ml of enzyme solution for reaction; *C* is corresponded to the value on the p-nitrophenol optical density curve.

### 2.6. Chemical composition and fermentation characteristics analysis

To analyze fermentation characteristics of silages, 20 g wet samples were taken and homogenized in 180 mL sterile water at 4°C for 24 h (Arshad et al., 2020). The mixture was then filtered through rapid qualitative filter paper (Fushun Civil Affairs Filter Paper Factory, Liaoning, China) and centrifuged at 3000 r/min for 10 min. The supernatant was collected for the following analysis. The pH was measured by pH meter (pHS-3C, Shanghai Instrument & Electronics Science Instrument Co., Ltd., Shanghai, China). The ammonia nitrogen (AN, g/kg TN) content was determined by using the phenol-hypochlorite colorimetric method (Xia et al., 2018). The contents of lactic acid (LA), acetic acid (AA), propionic acid (PA) and butylic acid (BA) were analyzed on gas chromatography (Fuli 9790II, Zhejiang Fuli Analytical Instrument Co., Ltd., Wenzhou, China.) (Ding et al., 2019).

The rest silage samples were dried at 105°C for 30 min and then at 65°C for 48 h to measure the DM content. Prior to the chemical analysis, the dried samples were ground into powder with grinder through a 2 mm screen. Specifically, the water-soluble carbohydrate (WSC) was extracted by using anhydrous ethanol and analyzed using the anthrone method. Total nitrogen (TN), ether extract (EE) and crude ash (Ash) were determined according to the procedure described by Fountoulakis et al. (2017), and the CP was calculated as TN × 6.25. The contents of NDF (Neutral Detergent Fiber) and ADF (Acid Detergent Fiber) were determined by the methods of Van Soest (Chen et al., 2021).

### 2.7. Microbial community of silages

Total genomic DNA of surface bacteria of fresh and ensiled sorghum fermented for 0, 7, 15, 30, and 60 days were extracted using a DNA isolation kit. The DNA quality and quantity were assessed by the NanoDrop2000. Afterwards, DNA samples were stored at-80°C until used. The V3-V4 region of the bacterial 16S rRNA gene was amplified with the common primer pair (forward primer 799F, 5′-AACMGGATTAGATACCCKG-3′ and reverse primer 1193R, 5′-ACGTCATCCCCACCTTCC-3′) combined with adapter and barcode sequences. The PCR amplification was performed as follows: 4 μL buffer, 0.4 μL Q5 High-Fidelity DNA Polymerase, 10 μL High GC Enhancer, 2 μL dNTP, 0.8 μM of each primer, 10 ng genome DNA, and sterilized deionized water were mixed to make the total volume to 20 μL. The fungus ITS gene was amplified with the common primer pair (forward primer ITS1F, 5′-CTTGGTCATTTAGAGGAA GTAA-3′ and reverse primer ITS2R, 5′-GCTGCGTTCTTCATCGATGC-3′) combined with adapter and barcode sequences. The PCR amplification was performed as follows: 10 μL Taq, 0.8 μM of each primer, 10 ng/μL Template DNA, and sterilized deionized water were mixed to make the total volume to 20 μL. Thermal cycling conditions included: an initial denaturation at 95°C for 3 min, followed by 27 cycles (95°C for 30 s, 50°C for 30 s and 72°C for 45 s) and a final extension at 72°C for 10 min. The original sequences were processed by Meiji Bio Cloud Platform. The RNA-Seq reads were deposited and available at the database Sequence Read Archive at NCBI (PRJNA921848).

### 2.8. Data analysis

The data were expressed as “mean ± standard deviation.” One-way ANOVA was applied to analyze the influence of additives on HCN contents and the parameters of silage quality using SPSS 23.0 (SPSS Inc., Chicago, IL, USA). Significance was tested using Tukey’s multiple range test at *p* < 0.05. The figures were made using origin 2021 (Northampton, MA, USA). After comparison with the NCBI 16S rRNA database (classified at a bootstrap threshold of 0.8) using RDP classifier software, sample ordination based on beta diversity was examined using principal coordinate analysis (PCoA) with phylogeny-based (UniFrac) unweighted and weighted distance. Heat map analysis was used to confirm the relationship between the microbial species, fermentation quality and HCN contents in ratooning sorghum silage. The high throughput sequencing data were analyzed on the online platform of Majorbio Cloud Platform.^1^

## 3. Results

### 3.1. Agronomic characters, nutritional quality and HCN content of ratooning sorghum

After two months’ regrowth, the ratooning sorghums were in their heading and flowering stages, with plant height reaching 157.56 cm, stem diameter 1.77 cm, and stem/leaf ratio 4.45 in year 2020 (Table 1). Similar results were also observed in year 2021. The yield on DM base reached 13.5 t/ha in 2020 and 12.78 t/ha in 2021 for ratooning sorghum. The contents of CP reached 6.27% in 2020 and 7.0% in 2021, and a higher content of WSC was also observed in ratooning sorghum. However, the contents of HCN in ratooning sorghum reached 892 mg/kg FW in 2020 and 801 mg/kg FW in 2021.

**Table 1:** Agronomic traits and nutrient contents of ratooning sorghum across 2 years.

Parameters	2020	2021
Plant height/cm	157.56 ± 1.58	160.43 ± 2.04
Stem diameter/cm	1.77 ± 0.60	1.75 ± 0.45
Number of blades	8.80 ± 0.33	8.46 ± 0.28
Stem leaf ratio	4.45 ± 0.55	4.77 ± 0.47
Yield (DM)/t/hm^2^	13.05 ± 0.78	12.78 ± 0.59
DM/%	16.34 ± 0.17	22.90 ± 0.09
CP/%DM	6.27 ± 0.03	7.00 ± 0.00
EE/%DM	6.53 ± 0.15	7.67 ± 0.32
NDF/%DM	65.71 ± 0.52	53.26 ± 0.26
ADF/%DM	37.79 ± 1.09	28.96 ± 1.27
Ash/%DM	13.05 ± 0.95	8.64 ± 0.18
WSC/%DM	24.81 ± 1.42	24.75 ± 4.50
HCN/mg/kg Fresh weight	891.99 ± 3.28	801.41 ± 0.52

DM, dry matter; CP, crude protein; EE, ether extract; NDF, neutral detergent fiber; ADF, acid detergent fiber; Ash, crude ash; WSC, water soluble carbohydrate; HCN, hydrogen cyanide.

### 3.2. Effects of silage on HCN content of ratooning sorghum

**Figure 1:**
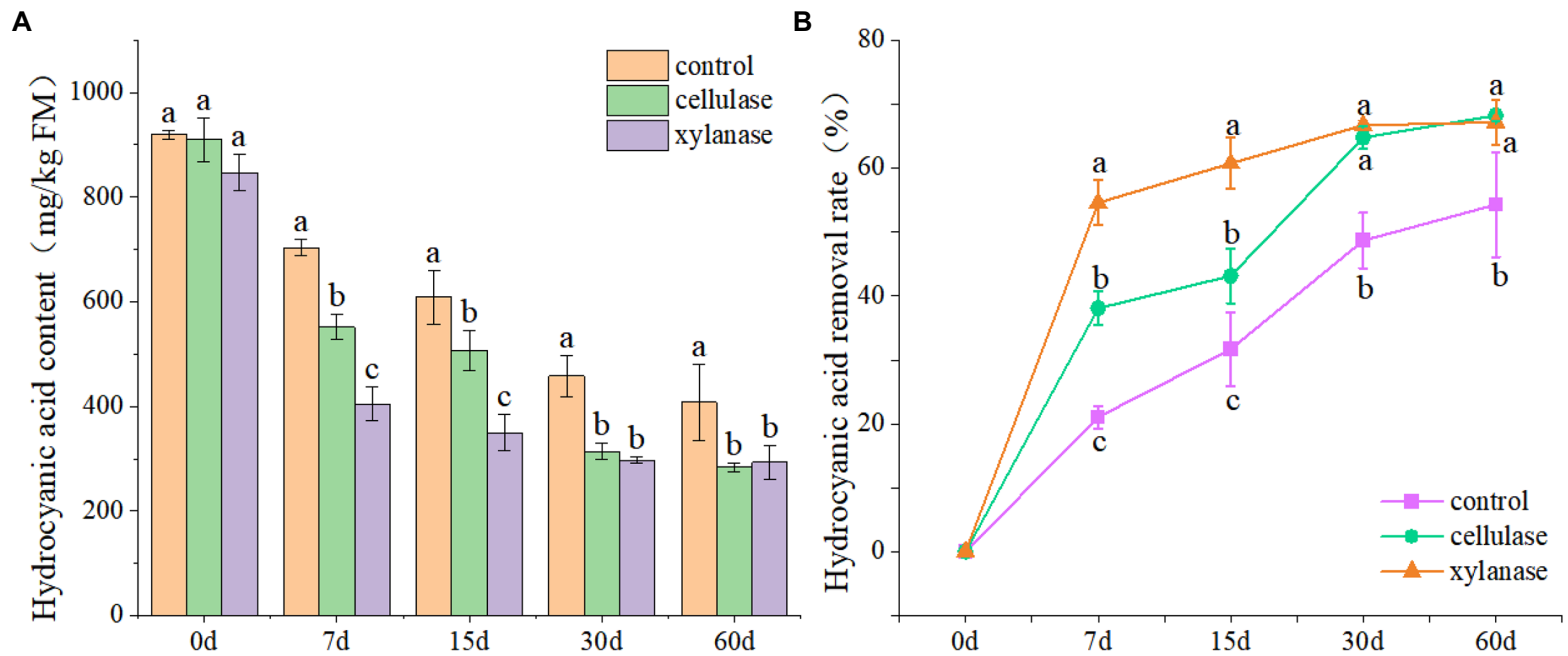
Hydrogen cyanide contents **(A)** and removal rate **(B)** of ratooning sorghum ensiled with different additives. CK, control; C, cellulase; X, xylanase. Different lowercase letter above the data bar represented significance between concentrations of *A. niger* within same fermentation time at *p* < 0.05.

In order to evaluate the effects of ensiling on the removal of CNglcs, the ratooning sorghum was ensiled with cellulase or xylanase. The results showed that the contents of HCN decreased significantly with the prolongation of fermentation (*p* < 0.05), and the lowest was observed 60 days after ensiling (Figure 1A). Overall, the contents of HCN reduced from 892 mg/kg FW at the beginning to 407 mg/kg FW 60 days after ensiling. Interestingly, lower contents of HCN were observed for silage ensiled with the additions of cellulase or xylanase (Figure 1B). However, the HCN contents in those silages were still above 200 mg/kg FW 60 days after ensiling, higher than the safety level. An increase of HCN removal rate was also observed with the prolongation of fermentation; however, the removal rate became slower 30 days after ensiling.

### 3.3. Effects of *A. niger* on HCN remover rate

**Figure 2:**
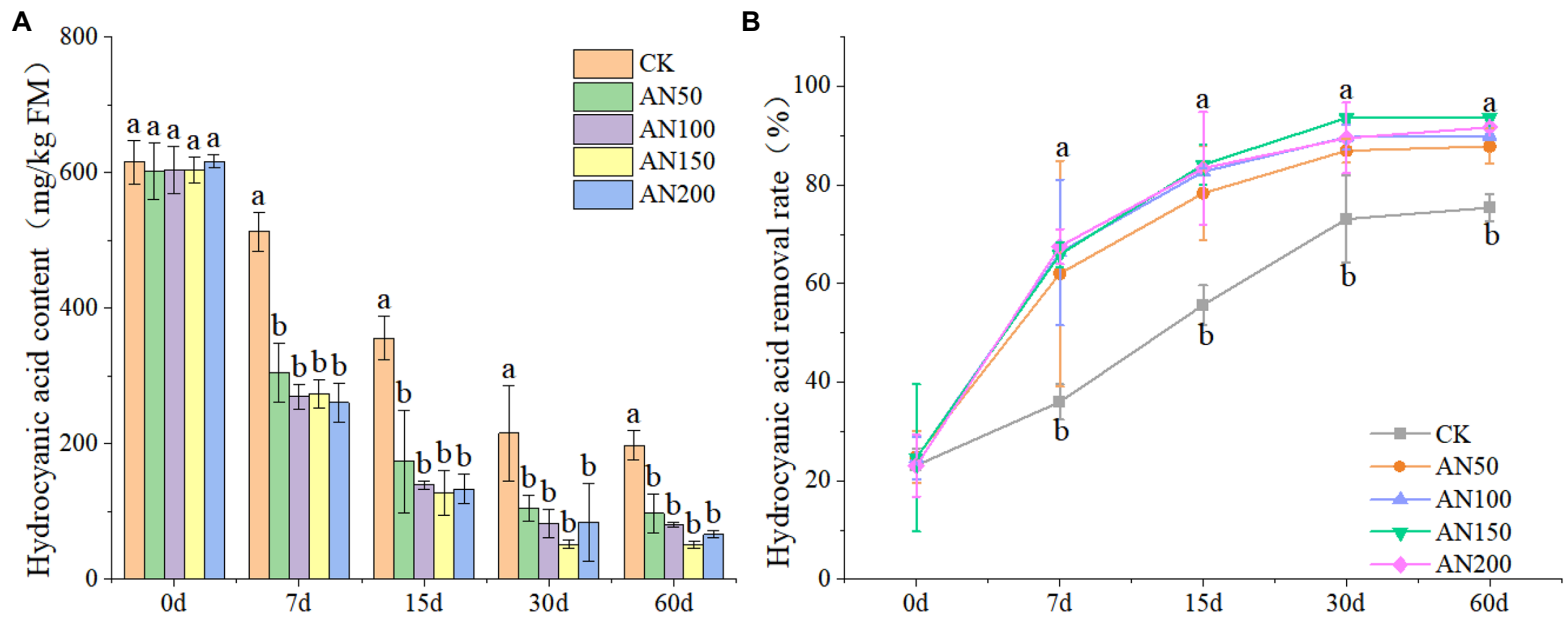
Hydrogen cyanide contents **(A)** and removal rates **(B)** of ratooning sorghum ensiled with different concentrations of *A. niger*. CK: 200 mL of sterile water per 3 kg of silage; AN50: 50 mL *A. niger* + 150 mL sterile water per 3 kg of silage; AN100:100 mL *A. niger* + 100 mL sterile water per 3 kg of silage; AN150: 150 mL *A. niger* + 50 ml sterile water per 3 kg of silage; AN200: 200 mL *A. niger* per 3 kg of silage. Different lowercase letter above the data bar represented significance between concentrations of *A. niger* within same fermentation time at *p* < 0.05.

In this study, the addition of *A. niger* significantly reduced the contents of HCN in ensiled ratooning sorghum (Figure 2A). The removal rate of HCN 60 days after ensiling reached 87.83–93.63% for silages ensiled with different concentrations of *A. niger*. However, for silage without *A. niger,* the removal rate was only 75.83% (Figure 2B). After 60 days of ensiling, the HCN contents in silage ensiled with different concentrations of *A. niger* were all lower than 120 mg/kg.

Though the HCN removal rate increased with the increase of fermentation time, the highest HCN amount removed from silage was observed at Day 7 for silages added with *A. niger*. For example, the HCN removal rates were all larger than 64% for silages added with *A. niger*, which were significantly higher than that of silage without *A. niger* (35.99%). The HCN removal rate did not increase with the increase of the concentrations of *A. niger*. Among the concentrations of *A. niger*, 150 ml *A. niger* + 50 ml sterile water per 3 kg was the optimal concentration for ratooning sorghum, with HCN removal rate reaching 93.63%.

### 3.4. Mechanisms of *A. niger* in degrading CNglcs

**Figure 3:**
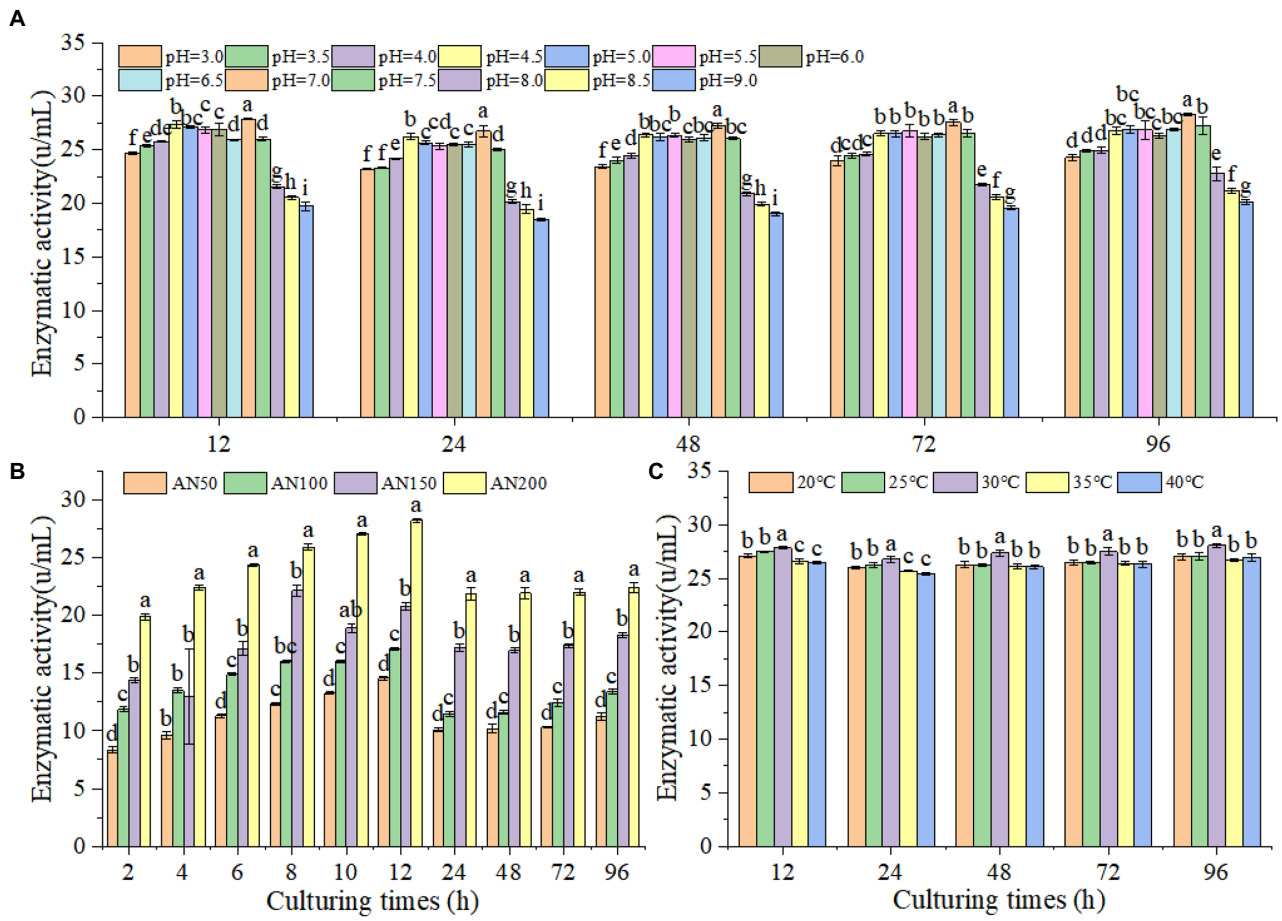
Activities of *β*-glucosidase produced by *A. niger* at different pH level **(A)**, inoculation amounts **(B)** and temperature **(C)**. Different lowercase letter above the data bar represented significance between pH values (concentrations or temperatures) within same culturing time at *p* < 0.05.

In order to prove whether *A. niger* could produce *β*-glucosidase under lower pH levels in ensiled ratooning sorghum, the *β*-glucosidase production characteristics of *A. niger* were measured under different inoculation amounts, pH levels and temperatures (Figure 3). The results indicated that *β*-glucosidase production increased with the increased inoculation amounts (Figure 3B). With the prolongation of culturing, *β*-glucosidase production increased first and reached the highest at 12 h, followed by a decrease at 24 h, and then kept stable from 24 to 96 h. *A. niger* could produce *β*-glucosidase at all tested pH levels from 3 to 9, suggesting that *A. niger* had the ability to produce *β*-glucosidase under the conditions of low silage pH levels (Figure 3A). However, the efficiency of *β*-glucosidase production depended upon pH levels, with relatively higher productions were obtained at 7.0. Interestingly, *A. niger* were more adaptable to acidic environments, and the enzyme production significantly decreased in alkaline environments, with the lowest productions were observed when the pH level was larger than 8. Considering the temperatures affecting silage fermentation, the *β*-glucosidase productions were measured under temperatures ranging from 20 to 40°C (Figure 3C). However, no significant difference in enzyme production could be observed at different temperatures.

### 3.5. Nutrient and fermentation qualities of ensiled ratooning sorghum

**Figure 4:**
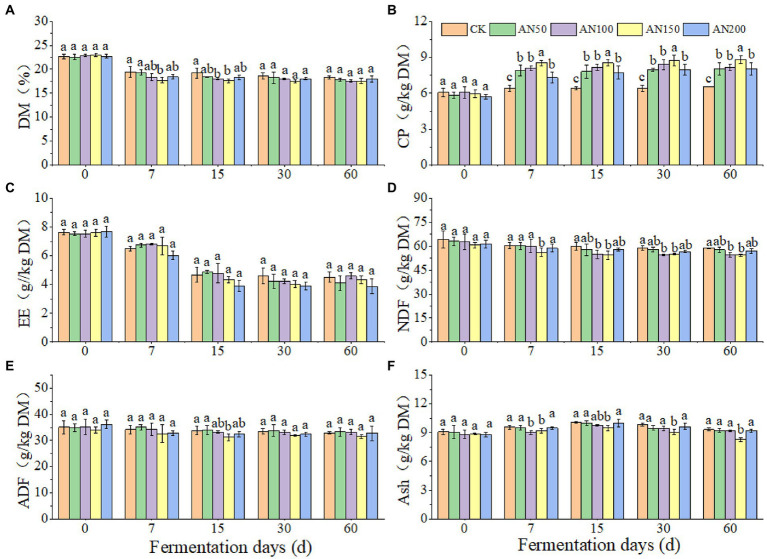
Nutrients of ratooning sorghum ensiled with different concentrations of *A. niger*. **(A)** dry matter (DM); **(B)**, crude protein (CP); **(C)**, ether extract (EE); **(D)**, neutral detergent fiber (NDF); **(E)**, acid detergent fiber (ADF); **(F)**, ash. CK: 200 mL of sterile water per 3 kg of silage; AN50: 50 mL *A. niger.* + 150 mL sterile water per 3 kg of silage; AN100:100 mL *A. niger.* + 100 mL sterile water per 3 kg of silage; AN150: 150 mL *A. niger.* + 50 mL sterile water per 3 kg of silage; AN200: 200 mL *A. niger.* per 3 kg of silage. Different lowercase letter above the data bar represented significance between concentrations of *A. niger.* within same fermentation time at *p* < 0.05.

**Figure 5:**
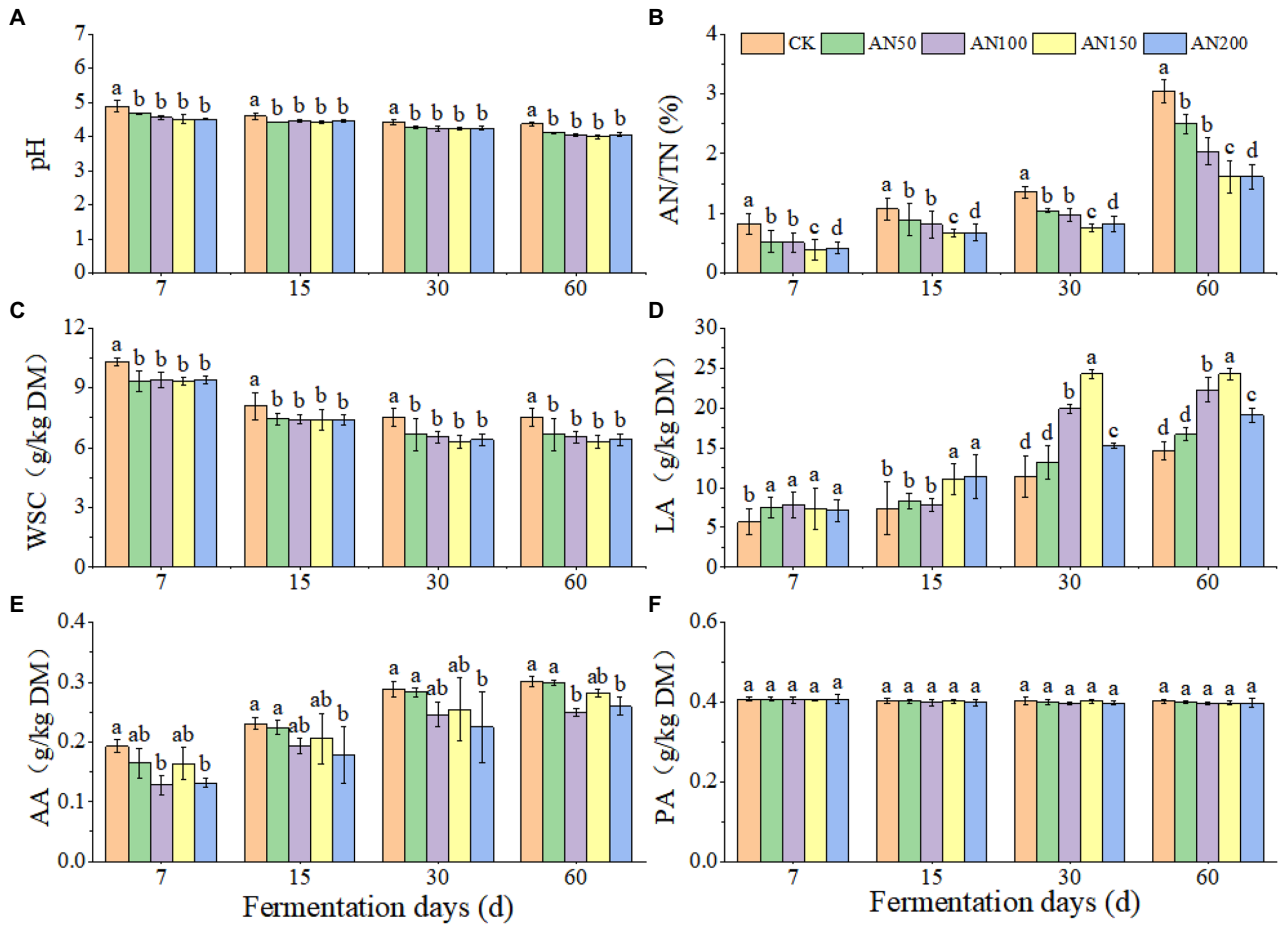
Silage quality of ratooning sorghum ensiled with different concentrations of *A. niger*. **(A)**, pH; **(B)**, ammonia nitrogen (AN); **(C)**, water soluble carbohydrate (WSC); **(D)**, lactic acid (LA); **(E)**, acetic acids (AA); **(F)**, propionic acid (PA). CK: 200 mL of sterile water per 3 kg of silage; AN50: 50 mL *A. niger.* + 150 mL sterile water per 3 kg of silage; AN100:100 mL *A. niger.* + 100 mL sterile water per 3 kg of silage; AN150: 150 mL *A. niger.* + 50 mL sterile water per 3 kg of silage; AN200: 200 mL *A. niger.* per 3 kg of silage. Different lowercase letter above the data bar represented significance between concentrations of *A. niger.* within same fermentation time at *p* < 0.05.

To check whether *A. niger* addition for CNglcs degradation was also efficient in obtaining higher quality silages, the nutrient and fermentation qualities of ensiled ratooning sorghum were also evaluated (Figures 4, 5). Compared with silage without *A. niger*, the CP contents in silage with *A. niger* increased significantly (*p* < 0.05) (Figure 4B), whereas the contents of DM and NDF showed a decreased trend (*p* > 0.05) (Figures 4A,D), with the variations of other nutrients depending upon fermentation time and *A. niger* concentrations (Figure 4). For example, EE contents reduced when the *A. niger* concentrations increased above 150 *A. niger* + 50 ml sterile water per 3 kg after15 days of fermentation (*p* < 0.05) (Figure 4C). Overall, the DM, EE and NDF decreased, whereas the CP increased with the prolongation of fermentation. Among the different concentrations of *A. niger*, higher CP content and lower NDF and ADF contents were observed for silage ensiled with AN100 and AN150. Meanwhile, the pH and AN/TN in silage with *A. niger* was lower whereas the LA concentration was higher than those without adding *A. niger* (*p* < 0.05) (Figure 5). No significant difference was observed for the concentrations of AA and PA in silages.

### 3.6. Bacterial community diversity and compositions in ratooning sorghum silage

Using high-throughput sequencing, the bacterial community was analyzed. The coverage values of all samples were above 99%, indicating that most bacteria were detected. With the prolongation of fermentation, the number of OUT, index of Shannon, ace and chao reduced, while Simpson index increased (Table 2), resulting in a decrease of the bacterial biodiversity in ratooning sorghum silage. The silage ensiled with *A. niger* decreased the alpha diversity before ensiling (48 h was given for propagation under oxygen before sealing).

**Table 2:** Alpha diversity in ratooning sorghum ensiled with *A. niger* for different days.

Items	Day 0		Day 7		Day 15		Day 30		Day 60	
	Control	*A. niger*	Control	*A. niger*	Control	*A. niger*	Control	*A. niger*	Control	*A. niger*
OTU	1,546 ± 47.28a	1,446 ± 31.75b	610 ± 38.79b	674 ± 15.72a	518 ± 81.03b	591 ± 18.23a	352 ± 20.66a	375 ± 12.86a	316 ± 27.43b	484 ± 33.38a
Shannon	4.16 ± 0.09a	3.79 ± 0.11b	2.30 ± 0.10b	2.67 ± 0.06a	2.66 ± 0.06a	2.95 ± 0.32a	1.42 ± 0.24a	1.47 ± 0.03a	1.31 ± 0.21b	2.047 + 0.08a
Simpson	0.06 ± 0.00b	0.10 ± 0.02a	0.27 ± 0.02a	0.18 ± 0.01b	0.11 ± 0.01b	0.18 ± 0.02a	0.45 ± 0.01a	0.44 ± 0.02a	0.40 ± 0.00a	0.26 ± 0.02b
Ace	1717 ± 48.75a	1,623 ± 13.41b	750 ± 37.02b	824 ± 30.69a	654 ± 26.03b	722 ± 31.45a	447 ± 16.06b	469 ± 32.72a	398 ± 51.07b	574 ± 25.83a
Chao	1,650 ± 55.20a	1,555 ± 13.41b	729 ± 37.47b	800 ± 33.98a	637 ± 29.62b	681 ± 34.90a	436 ± 16.95a	456 ± 27.30a	387 ± 47.14b	554 ± 26.21a
Coverage	0.997 ± 0.00	0.997 ± 0.00	0.998 ± 0.00	0.998 ± 0.00	0.999 ± 0.00	0.999 ± 0.00	0.999 ± 0.00	0.999 ± 0.00	0.999 ± 0.00	0.999 ± 0.00

Different lowercase letter after the data represented significance between concentrations of *A. niger* within same fermentation time at *P* < 0.05.

**Figure 6:**
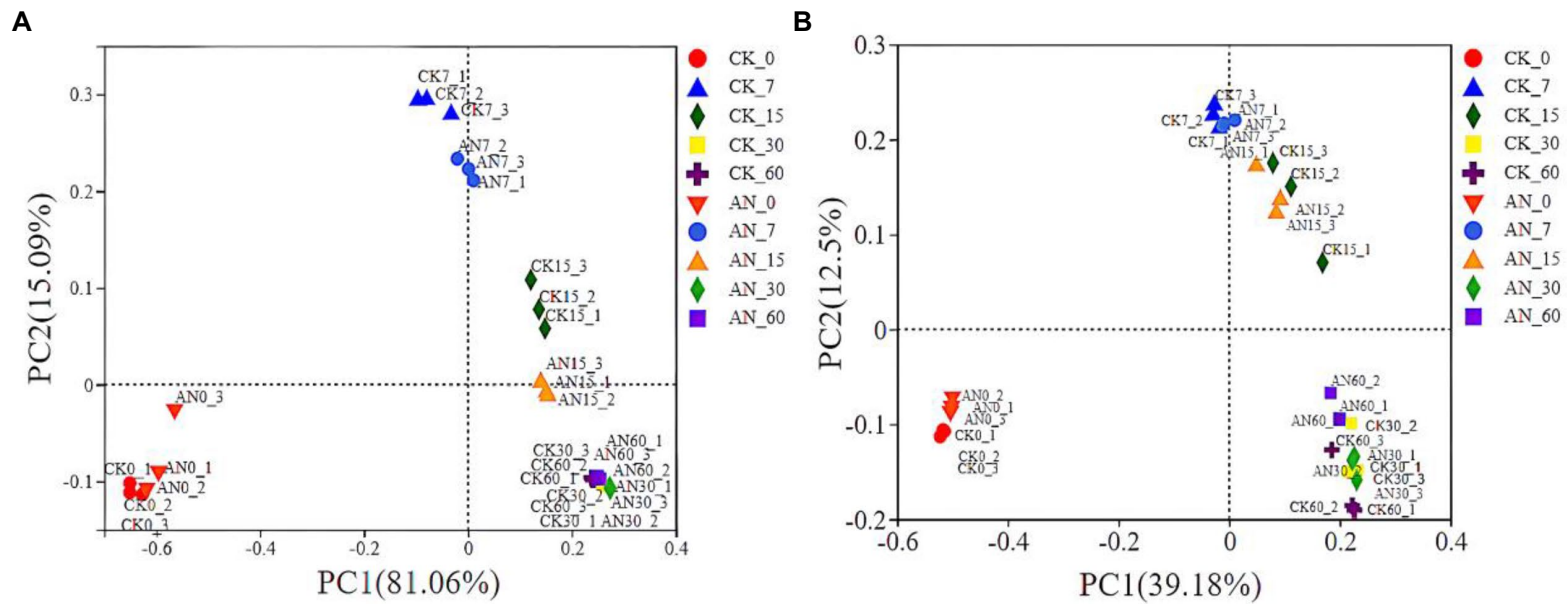
The community dissimilarities calculated by unweighted **(A)** and weighted **(B)** UniFrac distances. CK, control; AN, with *A. niger*.

Based on the community dissimilarities calculated by unweighted and weighted UniFrac distances, principal coordinates analysis (PCoA) indicated that the bacterial species had a significant succession with the prolongation of fermentation time (Figures 6A,B). However, the bacterial communities between silages with and without *A. niger* showed no obvious difference in succession. In PCoA analysis, the microbial diversities were grouped into three clusters, 0 day, 7 and 15 days, and 30 and 60 days. Overall, the microbial diversity of silage at 0 day was obviously separated from other silages.

**Figure 7:**
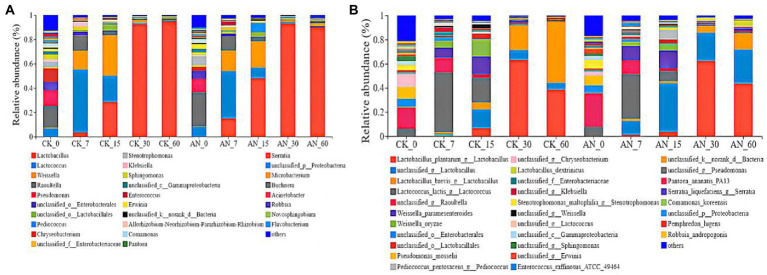
Relative abundances of ratooning sorghum silage bacterial based on genus **(A)** and species **(B)**. CK, control; AN, with *A. niger*.

The silage at day 7 was dominated by Lactobacillus, Lactococcus, Weisseria and Raoultella (Figure 7A). The dominating species were *L. plantarum*, Lactococcus_lactis, Weissella_paramesenteroides and unclassified_Raoultella (Figure 7B). Higher relative abundance of Lactococcus (39.11–51.67%) and lower relative abundance of Lactobacillus (28.75–30.06%) were observed during early fermentation period. However, with the fermentation continued, the pH declined, Lactobacillus (48.08%) became the dominant bacteria at day 15, which became more active when the pH decreased. From 30 to 60 days of fermentation, Lactobacillus dominated the whole silage process; and the dominant species were L. plantarum, L. brevis, L. lactis, L. unclassified and L. dextrinicus (Figure 7B). Among these LAB species, L. plantarum and L. brevis dominated the microbial community after 60 days of fermentation.

Compared with the control, the silage added with *A. niger* increased the abundance of *L. unclassified* while decreased the abundance of *L. lactis*, *Lactococcus* and *Weissella-oryzae* from 7 to 15 days of fermentation. However, the abundance of *L.unclassified* and *Lamellae* increased while the abundance of *L. brevis* and *L. dextrinicus* decreased from 30 to 60 days of fermentation.

### 3.7. Correlation analysis between microorganism and fermentation characteristics

**Figure 8:**
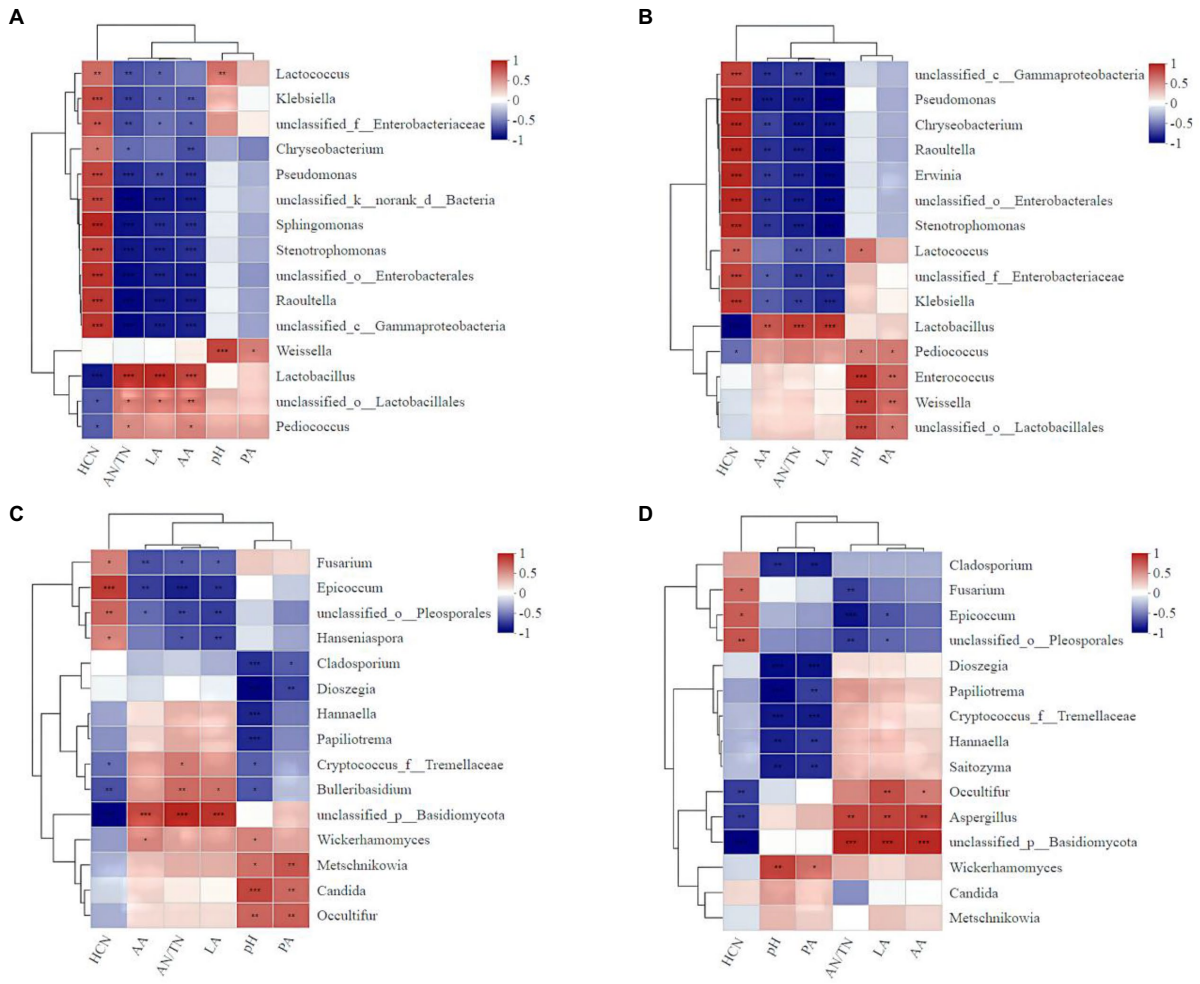
Heat map of the correlations between bacterial (fungal) species and the fermentation characteristics and the HCN content of ratooning sorghum silage. Fermentation characteristics and HCN content are displayed horizontally and the top 15 bacterial species were displayed vertically. The corresponding value of the middle heat map is the Spearman correlation coefficient *r*, the legend on the right is the color distinction of different *r* values; *r* < 0 indicates a negative correlation (blue), *r* > 0 indicates a negative correlation (red), and ‘*’, ‘**’, and ‘***’ represent *p* < 0.05, *p* < 0.01, and *p* < 0.001, respectively. **(A)**, bacterial of control; **(B)**, bacterial with *A. niger* addition; **(C)**, fungal of control; **(D)**, fungal with *A. niger* addition.

The addition of *A. niger* significantly influenced the correlations between microbe abundance and the silage qualities (Figure 8). For example, the relative abundances of *Lactococcus* (*r* = 0.65), *Enterobacter* (*r* = 0.58), *Enterococcus* (*r* = 0.82), *Weigella* (*r* = 0.82) and *Lactobacillus* (*r* = 0.81) in silage with *A. niger* were positively correlated with the pH value (Figure 8A). However, for silage without *A. niger*, only the relative abundance of *Lactococcus* (*r* = 0.68) and *Weigella* (*r* = 0.83) were positively correlated with pH value (Figure 8B). For both silages with and without *A. niger*, AN/TN ratio was negatively correlated with the abundance of *Lactococcus* (*r* = −0.65). The HCN contents in silage added with *A. niger* (*r* = −0.67) was negatively correlated with the abundance of *A. niger* (Figure 8D), and the abundance of *Lactobacillus* and *Pediococcus* (Figures 8A,B).

## 4. Discussions

When facing a forage shortage, increasing the forage resources can meet the requirement of increasing demand. Our results indicated that the ratooning sorghum has the potential to be used as forages. When compared with other annual forages, the yield on DM base reached 13.5 t/ha in 2020 and 12.78 t/ha in 2021 for ratooning sorghum, which was lower than that of forage sorghum (Yang and Lan, 2022) and silage corn (Na et al., 2022), but was higher than that of ryegrass and oat (Van Die and Entz, 2022). However, when compared with the growing period of other annual forages, the ratooning sorghum could provide forage without preparing the soil and seeding and without occupying principal food production season. This greatly reduced the cost of forage production and maximized the output from limited crop land, contributing to food safety (Xu et al., 2020). The chemical analysis further indicated that the contents of CP and WSC was similar with that of silage corn (Liu et al., 2021) and forage sorghum (Yang and Lan, 2022), implying that the ratooning sorghum might be a good alternative for fodder.

Generally, sorghum plants contain certain amounts of CNglcs, with its contents depending upon cultivars, soil water conditions, and growing stages (Sun et al., 2018). In this study, the contents of HCN in ratooning sorghum were greater than 800 mg/kg FW, significantly higher than the safety threshold (200 mg/kg FW). As one of the anti-nutritive, the CNglcs in ratooning sorghum increased its risk of poisoning when used as livestock feeds (Cressey et al., 2013). Therefore, the removal of CNglcs is the prerequisites for ratooning sorghum to be used as livestock feeds.

Ensiling could not reduce the HCN to safety level for ratooning sorghum. Interestingly, lower contents of HCN were observed for silage ensiled with the additions of cellulase or xylanase, suggesting that cellulase or xylanase addition would obtain higher HCN removal rate. Such positive effects of these silage additives were also reported on silage qualities (Thomas et al., 2013). However, the HCN contents in those silages 60 days after ensiling were still above 200 mg/kg FW, higher than the safety level. This suggested that these common silage additives could not completely remove HCN from ensiled ratooning sorghum. With the prolongation of fermentation, an increase of HCN removal rate was also observed, with the removal rate became slower 30 days after ensiling. Though no evidence has reported that the pH level might influence the HCN removal rate of silage, it seems that HCN removal rate reduced with the decrease of silage pH levels. Therefore, selecting silage additive that can degrade CNglcs at lower pH level might be a way to remove the HCN further.

*A. niger*, the most abundant mold found in the environment, has been shown to be effective in improving the silage qualities of rice straw and Napier grass (Bureenok et al., 2019; Li et al., 2022). And the safety and efficacy of the feed additives containing cellulase and endo-1,3(4)-*β*-glucanase produced by *A. niger* were also evaluated, proving their potential to be used as silage material (Rychen et al., 2018). A study by Liu et al. (2010) reported that inoculation of *A. niger* had a notable role on the removal of cyanoside of rubber seed meal. The HCN contents in silage ensiled with different concentrations of *A. niger* were all lower than 120 mg/kg after 60 days, suggesting that the addition of *A. niger* was effective in degrading CNglcs, with the remaining HCN in silage meeting the requirements of livestock safety. The highest HCN amount removed from silage was observed at Day 7 for silages added with *A. niger,* indicating that the *A. niger* might mainly degrade CNglcs at early days of silage fermentation. For an aerobic microbe, *A. niger* cannot adapt to the anaerobic condition of fermentation process (Meijer et al., 2007). Therefore, the positive effect of *A. niger* on HCN removal might mainly rely on the oxygen remained in silage during the early days of fermentation. During the late fermentation period, the *A. niger* would die, reducing their abilities to produce enzymes to degrade CNglcs. Overall, for ratooning sorghum, 150 ml *A. niger* + 50 ml sterile water per 3 kg was the optimal concentration, with HCN removal rate reaching 93.63%. This suggested that suitable concentration of *A. niger* should be screened to efficiently degrading CNglcs in silages.

Generally, CNglcs are degraded by enzyme to produce HCN (Cressey and Reeve, 2019). *β*-glucosidase, enzyme from the source plant material, has been shown to be involved in catalyzing the sequential breakdown of CNglcs in cyanogenic plants (Bhalla et al., 2017). *A. niger* had the ability to produce *β*-glucosidase under the various pH levels (3 to 9) and temperatures (20°C to 40°C), contributing to the degradation of CNglcs. A study on *Prunus armeniaca* has also shown that the *β*-glucosidase exhibited higher activities under low pH levels (4 to 6), and the optimum temperature for the activity of this enzyme was 35°C (Bhalla et al., 2017). Oriente et al. (2015) also reported that the optimum pH for *β*-glucosidase activity was 4.0 although it was stable in the range of 4.0–7.0. Therefore, the environmental conditions of the ensiling ratooning sorghum, lower pH levels in silage and the suitable temperature of fall, might be helpful to remove the CNglcs, and finally resulting in a safe HCN content for livestock. Overall, the addition of *A. niger* increased the contents of CP and LA, and decrease the contents of DM、NDF、pH and AN/TN in ratooning sorghum silage, thus decreasing the forage quality. The silage ensiled with 150 mL of *A. niger* + 50 mL of sterile water had the lowest pH, AN/TN and highest LA concentration. There are varieties of factors influencing the hydrolysis of plant protein during ensiling process (Xie et al., 2021). *A. niger* might influence the AN/TN through altering the distributions of microbes and thus the enzymes involved in protein hydrolysis (Guo et al., 2015). These results indicated that the addition of *A. niger* would increase the contents of CP and LA, and decrease the contents pH and AN/TN in ratooning sorghum silage, thus increasing the forage qualities. Li et al. (2022) also reported that the quality of mixed straw silage was depended upon concentrations of *A. niger*, and 100 mL of *A. niger* + 100 mL of sterile water was recommended to ensile rice straw. Higher concentrations of *A. niger* might induce competition between *A. niger* populations for oxygen and other nutrients for proliferation (Tian et al., 2018), reducing their efficiency to produce enzymes. Therefore, suitable *A. niger* concentration was also important to obtain higher quality silages.

Alpha diversity is mainly used to reflect species richness, evenness and sequencing depth (Caporaso et al., 2012). An decrease of the bacterial biodiversity in ratooning sorghum silage indicated that low pH level and low oxygen contents in silage might be not suitable for most phyllosphere microbes (Bashir et al., 2022), resulting in the propagation of microbes suitable under silage anaerobic conditions (Jung et al., 2022), like lactic bacterial. Adding *A. niger* decreased the alpha diversity before ensiling. This might be attributed to the quick increase of *A. niger* population under aerobic condition, which relatively reduced the bacterial diversity. However, with the extension of fermentation, the silages with *A. niger* showed higher bacterial diversity than that without *A. niger.* On one hand, most *A. niger* might die when the oxygen was depleted. On the other hand, the removal of CNglcs might benefit the growth of bacteria suitable under lower pH environments, increasing the bacterial diversity. PCoA analysis indicated that the bacterial species had a significant succession with the prolongation of fermentation time but showed no obvious difference in succession between silages with and without *A. niger*, suggesting that the addition of *A. niger*, overall, had no significant influence on ensiling microbe community.

Most of the bacteria involved in silage fermentation belong to *Lactobacillus*, *Laminaria*, *Weisseria* and *Leuconostoc* (Ni et al., 2018). A survey by Hasanuzzaman et al. (2014) also reported that in the early stage of alfalfa fermentation, *P. pentosus* was the dominant lactic acid bacteria (LAB), whereas *Lactobacillus* was dominant 7 days after silage. In this study, we also found that the dominant LAB species changes with the fermentation, and *L. plantarum* and *L. brevis* dominated the microbial community after 60 days of fermentation. These results indicated that the predominant microbes in silage of ratooning sorghum were similar with those of other sorghum silages (Tang et al., 2020; Wang et al., 2022; Zhang et al., 2022), suggesting that adding *A. niger* was feasible in silage fermentation for ratooning sorghum. However, some LAB species such as *L. lactis*, *Lactococcus* and *Weissella-oryzae* from 7 to 15 days of fermentation *and L. brevis* and *L. dextrinicus* from 30 to 60 days of fermentation, were negatively influenced by adding *A. niger.* The pH level and oxygen amount reduced with the prolongation of silage fermentation, which would significantly influence the propagation and enzyme productions of *A. niger* (Dong et al., 2014; Kanizai Saric et al., 2021), finally altering its influence of silage fermentation as well as the activities of other microbes.

The positive correlation between the silage pH value and the relative abundances of *Lactococcus*, *Enterobacter*, *Enterococcu*, *Weigella* and *Lactobacillus* in silage with *A. niger* suggested that the addition of *A. niger* altered the abundance of certain microbe species during fermentation, and thus the silage qualities. It is well known that *Lactococcus* (*Weisseria*, *Laminaria* and *Enterococcus*) starts LA fermentation at the early stage of silage (Jung et al., 2022). However, these strains are sensitive to low pH and cannot survive in an acidic environment (Ogunade et al., 2018). Therefore, *Lactobacillus* plays an important role when pH reduces at the later stage of silage, in ensuring good fermentation quality of silage (Cai et al., 1998). Interestingly, the abundance of *Lactobacillus* and *Pediococcus* was also negatively correlated with HCN contents, indicating that in the fermentation process of ratooning sorghum, these microbes might also have the abilities to remove CNglcs. For example, reduction of cyanide (97.17%) in cassava leaf was achieved under *Lactobacillus plantarum* fermentation (Terefe et al., 2022). Though these microbes exist in sorghum silages, lower removal rate of CNglcs in silage without adding *A. niger* suggested that their efficiency in removing CNglcs was less than that of *A. niger.*

## 5. Conclusion

Our results indicate that the ratooning sorghum which contain higher amount of CNglcs can be used as safe animal feeds though ensiling with *A. niger*. The addition of *A. niger* reduced the HCN contents of ratooning sorghum silage during the early days of fermentation, which improved the nutritive qualities of ratooning sorghum silage and reduced the HCN contents under safety threshold. The addition of exogenous *A. niger* altered the microbial community and increased bacterial diversity during silage fermentation process. The *β*-glucosidase can be produced by *A. niger* in a wide pH and temperature ranges, showing its abilities to remove CNglcs under silage conditions. These results also provide the potential to utilize plants with high CNglcs as animal feeds in the future.

## Data availability statement

The datasets presented in this study can be found in online repositories. The names of the repository/repositories and accession number(s) can be found below: BioProject, PRJNA921848.

## Author contributions

JZ: investigation, software, data curation, formal analysis, and writing. BW: software, data curation, formal analysis, and writing. YS: investigation, methodology, visualization, and validation. JY, TW, and WZ: investigation, methodology, visualization, and validation. JZ: investigation, methodology, and visualization. CQ: investigation, methodology, and visualization. YG: conceptualization, data curation, funding acquisition, project administration, supervision, validation, and writing – review and editing. All authors contributed to the article and approved the submitted version.

## Funding

This work was funded by Chongqing Technology Innovation and Application Development Project (cstc2021jscx-gksbX0014), “First Class Grassland Science Discipline” program in Shandong Province, China, and Special Fund Project of Provincial Science and technology innovation development in agricultural high district of Yellow River Delta (2022SZX32).

## Conflict of interest

JZ was employed by Chongqing Jiangxiaobai Farm Co., Ltd.

The remaining authors declare that the research was conducted in the absence of any commercial or financial relationships that could be construed as a potential conflict of interest.

## Publisher’s note

All claims expressed in this article are solely those of the authors and do not necessarily represent those of their affiliated organizations, or those of the publisher, the editors and the reviewers. Any product that may be evaluated in this article, or claim that may be made by its manufacturer, is not guaranteed or endorsed by the publisher.

## Footnotes

^1^http://www.majorbio.com

## Abbreviations

AA: Acetic acid
ADF: Acid Detergent Fiber
AN: Ammonia nitrogen
Ash: Crude ash
BA: Butylic acid
CNglcs: Cyanogenic glycosides
EE: Ether extract
HCN: Hydrogen cyanide
LA: Lactic acid
LAB: Lactic acid bacteria
NDF: Neutral Detergent Fiber
PA: Propionic acid
PCoA: Principal coordinates analysis
PDA: Potato Dextrose Agar medium
PDB: Potato Dextrose Broph liquid medium
TN: Total nitrogen
WSC: Water-soluble carbohydrate
